# A prospective study comparing quantitative Cytomegalovirus (CMV) polymerase chain reaction in plasma and pp65 antigenemia assay in monitoring patients after allogeneic stem cell transplantation

**DOI:** 10.1186/1471-2334-6-167

**Published:** 2006-11-21

**Authors:** Giuseppe Gentile, Alessandra Picardi, Angela Capobianchi, Alessandra Spagnoli, Laura Cudillo, Teresa Dentamaro, Andrea Tendas, Luca Cupelli, Marco Ciotti, Antonio Volpi, Sergio Amadori, Pietro Martino, Paolo de Fabritiis

**Affiliations:** 1 Department of Cellular Biotechnology and Hematology, Univ. "La Sapienza", Rome, Italy; 2 Hematology and Clinical Pathology, Tor Vergata University, Rome, Italy; 3 Hematology, Tor Vergata University, S. Eugenio Hospital, Rome, Italy; 4 Department of Public Health, Tor Vergata University, Rome, Italy

## Abstract

**Background:**

Low levels of Cytomegalovirus (CMV) viral load are frequently detected following allogeneic stem cell transplantation (SCT) and CMV disease may still develop in some allogeneic SCT patients who have negative pp65-antigenemia (pp65-Ag) or undetectable DNA. Pp65Ag is a sensitive method to diagnose CMV infection. Quantitative CMV-DNA PCR assay in plasma has been proposed to monitor CMV infection in SCT patients. We evaluated the clinical utility of pp65Ag and PCR assay in plasma of SCT recipients.

**Methods:**

In a prospective longitudinal study, 38 consecutive patients at risk of CMV infection (donor and/or recipient CMV seropositive) were weekly monitored for CMV infection by both quantitative CMV-PCR in plasma (COBAS AMPLICOR CMV MONITOR) and pp65 Ag, during the first 100 days after SCT.

**Results:**

A total of 534 blood samples were simultaneously analysed for pp65Ag and PCR. Overall, 28/38 patients (74%) had active CMV infection within 100 days from SCT. In 16 patients, CMV was first detected by pp65 Ag alone; in 5 patients by both methods and in 6 by PCR assay alone; one patient had CMV biopsy-proven intestinal disease without pp65Ag and PCR assays positivity before CMV disease. Overall, three patients developed intestinal CMV disease (7.9%): one had negative both pp65Ag and PCR assays before CMV disease, one had disease and concomitant positivity of both methods, while in the remaining patient, only pp65Ag was positive before CMV disease.

**Conclusion:**

Plasma PCR(COBAS AMPLICOR CMV MONITOR) and pp65Ag assays were effective in detecting CMV infection, however, discordance between both methods were frequently observed. Plasma PCR and pp65Ag assays may be complementary for diagnosis and management of CMV infection.

## Background

Cytomegalovirus (CMV) infection still causes significant morbidity and mortality following allogeneic stem cell transplantation (SCT) [1]. The impact of this infection on transplantation extends beyond the direct clinical manifestations (e.g. pneumonitis, gastrointestinal diseases, hepatitis, marrow suppression) and includes indirect effects such as increased incidence of other opportunistic infections and decreased patient survival [2,3]. Therefore, prevention and treatment of active CMV infection must be based on sensitive and reliable diagnostic assays. Pre-emptive therapy administered on the basis of evidence of CMV reactivation has become a common strategy in the treatment of SCT recipients [4,5]. A commonly used test to detect active CMV infection is the immune-fluorescence staining of CMV lower matrix protein pp65 (UL83) in peripheral blood leukocytes (PBL) [6]. In the pp65 antigenemia (pp65Ag) assay, the number of CMV positive cells in the peripheral blood reflects the viral load and high numbers of pp65 positive cells correlate with CMV disease [7,8]. A significant threshold is used in transplant recipients for predicting CMV disease. Clinically relevant threshold of the number of infected PBL differs among the different patient populations. Thresholds of more than 10 positive cells/200.000 PBL and >1 or 2 positive cells/200.000 cells have been suggested to guide pre-emptive therapy in solid organ and SCT recipients, respectively [9,10]. Pre-emptive therapy based on pp65 antigen detection in PBL is associated with a reduction in the incidence of CMV disease in allogeneic SCT recipients [11]. However, the antigen based diagnostic test has some disadvantages: low sensitivity for detecting early active CMV infection or disease that may occur before engraftment due to the lack of leukocytes during the period of aplasia (requirement of neutrophil counts >0.5 × 10^9^cells/L) and low positive predictive value for the occurrence of CMV gastroenteritis [12,13]. Furthermore, pp65Ag assay requires processing of the blood samples within few hours, is time consuming, and cannot be automated.

PCR-based methods have been recently evaluated for diagnosing and monitoring CMV infection after allogeneic SCT [14-16]. However, it remains difficult to evaluate the exact role of the previously reported PCR assays in guiding pre-emptive CMV therapy in the allogeneic SCT recipients, due to differences in the origin of samples [whole blood, plasma, serum, PBL, peripheral blood mononuclear cells (PBMC)] and the type of PCR procedures (qualitative versus quantitative) used for monitoring [17-21].

Previous studies showed that the presence of CMV in plasma or serum is indicative of active viral replication being associated with a high predictive value for CMV disease in SCT recipients [22,23] and in HIV-infected patients [24]. In addition, plasma offers the opportunity to detect active CMV infection during periods of severe cytopenia when cell-based assays (PCR on leukocytes and pp65-antigenemia) perform poorly [25]. However, clinical results of plasma PCR in allogeneic SCT recipients are still controversial since CMV PCR assays used in clinical studies have been developed in-house [15,25] and the methods are not standardized.

There is much interest in the quantification of CMV load in blood for monitoring and prediction of CMV disease development and progression. Many studies have shown that the amount of CMV DNA is significantly associated with disease development [16,21,23]. However, while evidences indicate that high CMV load is associated with a higher risk of progression to CMV disease in solid organ transplant recipients, the association may be less clear for allogeneic SCT recipients [12]. Further, low levels of CMV viral load are frequently detected following allogeneic SCT [16,26] and CMV disease may still develop in some allogeneic SCT patients who have negative pp65-Ag or undetectable DNA [1].

In this study, 38 patients were prospectively monitored for CMV infection during the first 100 days post-SCT. Two different commercially available assays were simultaneously used to compare clinical utility of a cell-based pp-65 Ag test with plasma PCR (COBAS AMPLICOR CMV MONITOR). Pre-emptive therapy, based on a positive pp65Ag assay result, was administered in all SCT recipients.

## Methods

### Patients and study design

Between January 2003 and February 2005, 42 consecutive SCT recipients at risk for CMV infection (donor and/or recipient CMV seropositive) were included in the study. Four out of 42 patients were excluded because survived less than 50 days after transplantation without any evidence of pp65 Ag positive cells and/or CMV-DNA. Therefore, 38 patients were considered valuable for this study. Blood samples were tested for CMV by both quantitative CMV-PCR in plasma and pp65 Ag at weekly intervals and, on suspicion of CMV infection, from day -7 to day 100 post-transplant.

Patients underwent SCT at the Transplant Unit of S. Eugenio Hospital, Tor Vergata University, Rome, Italy. From the time of hospital admission onwards, patients were given standard prophylaxis for bacterial (levofloxacin 500 mg daily p.o.), and fungal (fluconazole at 200 mg twice daily p.o.) infections. Acyclovir at standard (250 mg/m^2 ^i.v. every 8 hours, or 200 mg four times a day) or high (500 mg/m^2 ^i.v. every 8 hours, or 800 mg p.o. four times a day) dose was given for prophylaxis of herpes simplex virus infections in related or unrelated transplants, respectively. No patients received prophylactic intravenous immunoglobulins, ganciclovir, foscarnet, or cidofovir. The ethic Committee of S. Eugenio Hospital, Tor Vergata University, Rome approved the study and an informed written consent was received from each patient. Patient characteristics are summarized in Table 1.

**Table 1:** Clinical characteristics

**Characteristics**	**Value**
Patients	38
Age, y	
Median	41
Range	(11–68)
Sex	
Male	22
Female	16
Diagnosis	
Acute leukemia	16
Lymphoma	6
Multiple myeloma	4
Myelodyspastic syndrome	4
Solid tumor	4
Non neoplastic disorders	3
Chronic lynphocytid leukemia	1
Acute GVHD	
0–I	27
II–IV	11
Total body irradiation	
Yes	15
No	23
Conditioning regimen	
Standard	25
Reduced intensity conditioning	13
Type of transplant	
Related	30
Unrelated	8
Hemopoietic reconstitution	
Median days of PMN >0.5 × 10^9^/L (range)	16 (11 – 41)
Median days of PLTS >20 × 10^9^/L (range)	15 (9 – 65)
Median days of PLTS >50 × 10^9^/L (range)	18 (11 – 88)
Source of stem cells	
Peripheral blood	31
Bone marrow	4
Cord blood	3
Prophylaxis of aGVHD	
CyA+MTX	25
CyA+MMF	6
CyA+PDN	4
Other	3
CMV status (donor/recipient)	
Positive/positive	26
Negative/positive	10
Positive/negative	2
Deaths	4

GVHD: Graft versus host disease; CyA: Cyclosporin-A; MTX: Methotrexate; PDN: Prednisone; CMV: Cytomeglovirus. MMF: Micofenolate Mofetil; PMN: Peripheral Mononuclear cells; PLTS: Platelets

### Criteria for the diagnosis of CMV infection and disease

Cytomegalovirus infection and disease were defined according to published recommendations [27]. Active CMV infection was defined as the detection of pp65 antigen in leukocytes and/or the presence of CMV DNA in plasma. CMV enteritis was defined as the presence of gastrointestinal symptoms, findings of macroscopic mucosal lesions on endoscopy, and demonstration of CMV infection (by culture, histopathologic testing and immunohistochemical analysis) in biopsy samples taken from colon. Detection of CMV by PCR alone was considered insufficient for the diagnosis of CMV intestinal disease.

### CMV serology

Serum samples were tested by a commercially available enzyme linked immunosorbent assay (Delta Biological, Italy), according to the manufacturer's instructions.

### Specimen processing

A 15 ml volume of EDTA-treated blood was collected from each patient. Ten ml were used for CMV Ag assay and processed within 4 hours; plasma obtained from the remaining blood was used for CMV PCR assay.

### Virological assays

#### Pp65 antigen test (Antigenemia assay)

The CMV pp65 antigenemia test was carried out with commercially available monoclonal antibodies, according to the standard protocol [CINA Kit, Argene]. Briefly, EDTA-treated whole blood samples were fractioned by dextran-sedimentation and lysis of erythrocytes. Slides were incubated with monoclonal anti-pp65 [pool of monoclonal antibodies (1C3+AYM-1), Biosoft, Paris, France]. The pp65 Ag results were reported as the total number of positive cells/200.000 PBL examined. All clinical decision regarding pre-emptive antiviral therapy were based on CMV Ag.

#### Quantification of CMV-DNA

CMV-DNA was quantified on plasma samples by using the Cobas Amplicor CMV Monitor, according to the manufacturer's instructions (Roche Molecular Systems). The dynamic range for quantification is approximately 3 log_10 _units, with 400 copies as the lower limit of detection.

### Preemptive therapy for prevention of CMV disease

Pre-emptive therapy was started at the first detection of pp65-Ag positive cells (≥ 1 positive cell/200,000 cells). Plasma CMV DNA results were not considered for clinical decision making. Antiviral pre-emptive therapy was based on intravenous infusion of either ganciclovir at 10 mg/Kg/day or foscarnet at 180 mg/Kg/day for 2 to 3 weeks followed by either ganciclovir at 5 mg/Kg/day or foscarnet at 90 mg/Kg/day for 2 to 3 weeks.

### Statistical analysis

The agreement between CMV detection assay was evaluated using the Kappa coefficient. Values of kappa above 0.75 indicate strong agreement; values between 0.40 and 0.75 represent fair to good agreement and value less than 0.40 reflect poor agreement [28].

The mean period of time between transplant and pp65 Ag or PCR positivity (whichever first occurred) were compared by the Student's t-test. Differences between median time of disappearance of pp65 Ag or DNA were tested using Mann-Withney test. After normalization of the pp65 Ag values by logarithmic transformation (log_10_), the correlation between the two test was evaluated with Pearson's correlation coefficient. Differences in percentages were tested using the Fisher's exact test (2-tailed). P-values less than 0.05 were considered statistically significant.

## Results

### Clinical outcome

Of the 38 valuable patients, 25 (70%) had active CMV infection (Table 2), 10 did not develop CMV infection and 3 developed pathologically diagnosed CMV colitis (Table 3).

**Table 2:** Patients with CMV infection

**UPN**	**ASSAY**	**DAYS FROM TRANSPLANT**															
		
		**-7**	**0**	**+7**	**+14**	**+21**	**+28**	**+35**	**+42**	**+49**	**+56**	**+63**	**+70**	**+77**	**+84**	**+91**	**+100**
**0119.1**	pp65 Ag	ND	ND	ND	- •	- •	- •	1•	-	-	-	- •	- •	- •	- •	- •	- •
	Plasma PCR	ND	ND	ND	- •	- •	- •	- •	-	-	-	- •	- •	- •	- •	- •	- •
**0120.1**	pp65 Ag	ND	- •	- •	- •	- •	7•	-	-	†							
	Plasma PCR	ND	- •	- •	- •	- •	970•	-	-	†							
**0122.1**	pp65 Ag	- •	- •	NE	- •	- •	- •	- •	4•	-	-	†					
	Plasma PCR	- •	- •	- •	- •	- •	- •	- •	- •	-	-	†					
**0123.1**	pp65 Ag	- •	- •	- •	- •	- •	- •	- •	1•	-	-	-	- •	- •	- •	- •	- •
	Plasma PCR	- •	- •	- •	- •	- •	- •	- •	- •	-	-	-	- •	- •	- •	- •	- •
**0124.1**	pp65 Ag	- •	- •	- •	- •	- •	4•	-	-	-	- •	1•	-	-	-	- •	- •
	Plasma PCR	- •	- •	- •	- •	- •	- •	-	-	-	- •	- •	-	-	-	- •	- •
**0125.1**	pp65 Ag	ND	- •	- •	- •	- •	- •	- •	- •	7•	-	-	-	- •	- •	- •	- •
	Plasma PCR	ND	- •	770•	- •	- •	- •	- •	- •	- •	-	-	-	- •	- •	- •	- •
**5244.2**	pp65 Ag	- •	- •	NE	- •	- •	- •	40•	2	80	7	-	-	-	ND	2•	-
	Plasma PCR	- •	- •	- •	- •	- •	- •	1980•	1500	10000	1010	-	-	-	ND	- •	-
**0127.1**	pp65 Ag	NE	NE	NE	NE	NE	- •	- •	20•	-	-	-	- •	- •	- •	- •	- •
	Plasma PCR	- •	- •	- •	- •	- •	- •	- •	- •	948	-	-	- •	- •	- •	- •	- •
**0128.1**	pp65 Ag	- •	- •	NE	- •	- •	- •	5•	-	-	-	- •	- •	- •	- •	- •	- •
	Plasma PCR	- •	- •	- •	- •	- •	- •	- •	-	-	ND	- •	- •	- •	- •	- •	- •
**0130.1**	pp65 Ag	- •	5•	-	-	-	- •	- •	- •	ND	- •	1•	-	-	-	- •	- •
	Plasma PCR	- •	-•	-	-	-	- •	- •	- •	ND	- •	- •	-	-	-	- •	- •
**0134.1**	pp65 Ag	- •	- •	NE	NE	- •	- •	40•	6	2	-	-	-	- •	- •	8•	-
	Plasma PCR	- •	- •	- •	- •	- •	- •	- •	-	-	-	-	-	- •	- •	- •	-
**0135.1**	pp65 Ag	- •	- •	NE	- •	- •	- •	- •	1•	-	-	-	- •	- •	- •	- •	- •
	Plasma PCR	- •	- •	- •	- •	- •	- •	- •	- •	-	-	-	- •	- •	- •	- •	- •
**0137.1**	pp65 Ag	- •	- •	NE	- •	- •	- •	- •	- •	5•	-	-	-	- •	- •	- •	- •
	Plasma PCR	- •	- •	- •	- •	- •	- •	- •	- •	- •	-	-	-	- •	- •	- •	- •
**0139.1**	pp65 Ag	- •	ND	- •	- •	- •	- •	- •	74•	30	6	-	-	-	ND	- •	- •
	Plasma PCR	- •	ND	- •	- •	- •	- •	- •	6490•	22400	9690	-	-	-	ND	- •	- •
**5224.2**	pp65 Ag	- •	- •	- •	- •	- •	- •	- •	30•	-	5	-	-	2	-	-	-
	Plasma PCR	- •	- •	- •	- •	- •	- •	550•	- •	-	825	-	-	-	-	-	-
**5298.1**	pp65 Ag	- •	- •	- •	- •	- •	- •	- •	- •	2•	-	-	-	- •	- •	- •	ND
	Plasma PCR	- •	- •	- •	- •	- •	- •	- •	- •	- •	-	-	-	- •	- •	- •	ND
**5176.1**	pp65 Ag	- •	- •	- •	- •	- •	- •	- •	- •	- •	60•	-	-	ND	- •	- •	- •
	Plasma PCR	- •	- •	- •	- •	- •	- •	- •	470•	3830•	NE	1400	-	ND	- •	- •	- •
**0149.1**	pp65 Ag	- •	- •	NE	- •	- •	- •	- •	- •	- •	- •	- •	- •	2•	-	NE	-
	Plasma PCR	- •	- •	-	- •	- •	- •	- •	- •	- •	- •	- •	- •	-	-	-	-
**0150.1**	pp65 Ag	- •	- •	- •	- •	- •	- •	- •	- •	10•	4	-	-	-	- •	- •	- •
	Plasma PCR	- •	- •	- •	- •	- •	- •	- •	- •	- •	832	1530	680	-	- •	- •	- •
**0151.1**	pp65 Ag	- •	- •	- •	- •	- •	5•	3	-	1	-	-	-	- •	- •	- •	- •
	Plasma PCR	- •	- •	- •	- •	- •	438•	-	695	-	-	-	-	- •	- •	- •	- •
**0152.1**	pp65 Ag	- •	- •	NE	ND	32•	-	144	-	13	30	3	-	-	-	- •	422•
	Plasma PCR	- •	- •	- •	ND	- •	568	8960	955	2670	1720	11110	3070	1250	659	- •	21200•
**0155.1**	pp65 Ag	- •	- •	- •	NE	- •	- •	ND	- •	- •	- •	- •	351•	9	-	-	-
	Plasma PCR	- •	- •	- •	- •	- •	- •	- •	463•	7860•	6510•	12600•	6790•	4770	-	-	-
**0164.1**	pp65 Ag	- •	- •	NE	NE	- •	- •	- •	- •	400•	4	7	-	-	-	- •	- •
	Plasma PCR	- •	- •	- •	- •	- •	- •	- •	891•	8280•	5830	7360	-	-	-	- •	- •
**5278.2**	pp65 Ag	- •	- •	NE	NE	- •	1•	1	-	-	-	- •	- •	- •	- •	- •	- •
	Plasma PCR	- •	- •	- •	- •	- •	- •	-	-	-	-	- •	- •	- •	- •	- •	- •
**0173.1**	pp65 Ag	NE	NE	NE	- •	- •	- •	ND	- •	- •	- •	- •	2•	-	-	-	- •
	Plasma PCR	- •	- •	- •	ND	- •	- •	- •	- •	- •	- •	1710•	1520•	-	-	ND	- •

UPN: unique patient number; Ag: antigenemia; PCR: polymerase chain reaction; ND: not done; NE: not evaluate; -: negative result; Ag and PCR values are expressed as N° of positive leucocytes or viral copies, respectively; †: death. Dots represent samples of patients not on pre-emptive therapy

**Table 3:** Patients with CMV disease

**UPN**	**ASSAY**	**DAYS FROM TRANSPLANT**															
		
		**-7**	**0**	**+7**	**+14**	**+21**	**+28**	**+35**	**+42**	**+49**	**+56**	**+63**	**+70**	**+77**	**+84**	**+91**	**+100**
**0133.1**	Pp65 Ag	- •	- •	- •	ND	- •	- •	- •	- •	- •	-** •	-	-	-	-	-	2
	Plasma PCR	- •	- •	- •	ND	- •	- •	- •	- •	- •	-** •	-	-	-	-	-	-
**0136.1**	pp65 Ag	- •	- •	- •	- •	- •	- •	- •	- •	400** •	83	1	5	2	-	4	-
	Plasma PCR	- •	- •	- •	- •	- •	- •	- •	- •	22600** •	12300	8810	6000	1290	-	-	-
**0138.1**	pp65 Ag	- •	- •	- •	7•	-**	-	-	- •	60- •	23	4	-	-	-	-	19
	Plasma PCR	- •	- •	- •	- •	-**	-	-	- •-	1230- •	4070	16700	2240	-	-	758	2250

UPN: unique patient number; Ag: antigenemia; PCR: polymerase chain reaction; ND: not done; NE: not evaluate; -: negative result; **: CMV disease. Dots represent samples of patients not on pre-emptive therapy.

All patients developed intestinal CMV disease and UPN 0136.1 died on day + 111 for CMV disease associated with aGVHD.

Four of the engrafted patients died within 100 days after SCT: two for progression of acute myeloid leukemia and colon carcinoma, respectively; one for acute GVHD and one for CMV disease associated with acute GVHD.

### Detection of CMV by pp65 antigenemia, and by the Cobas CMV PCR assays

Overall, 568 blood samples were analysed for pp65Ag and/or PCR; of the 35 (6.2%) samples obtained from patients in whom the pp65 Ag assay could not be performed because of the low number of cells (i.e. before engraftment or during severe neutropenia with absolute neutrophil counts of less than 200/μl), none was positive by plasma PCR assay. Of the 534 samples tested simultaneously for pp65 Ag and PCR, 31 (5.8%) were positive by PCR and pp65 Ag, 30 (5.6%) were pp65 Ag positive but PCR CMV negative, 22 (4.1%) were PCR positive/pp65Ag negative, and 451 (84.4%) were negative by both assays. CMV was detected in 83 samples (15%) by a single or both methods.

Relative sensitivities, specificities, and predictive values for detection of plasma DNA and pp65 Ag were calculated for both assays considering all specimens obtained from patients with and without anti-CMV therapy at the time of testing. Using as reference any positive test (either pp65 Ag or PCR assays), sensitivity/specificity/positive-predictive-value/negative-predictive-value for detection of CMV plasma DNA were 64%/100%/100%/94%, respectively. Using as reference any positive test (either pp65 Ag or PCR assays), sensitivity/specificity/positive-predictive-value/negative-predictive-value for detection of pp65 Ag were 73%/100%/100%/95%, respectively.

### Correlations between pp65 antigenemia and Cobas CMV PCR assays

**Figure 1:**
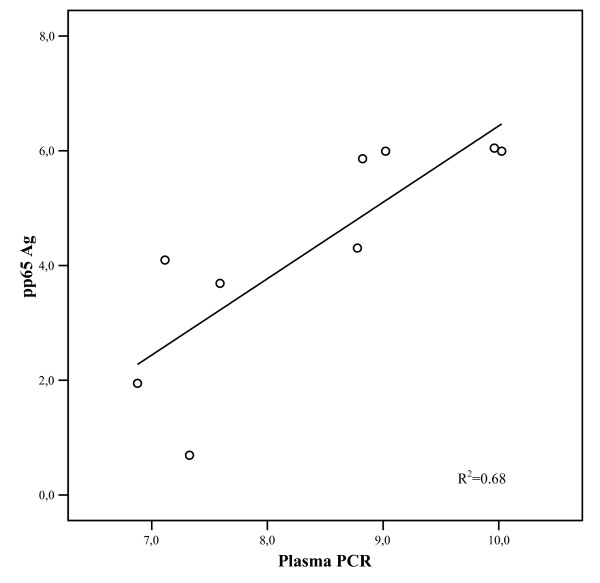
shows on a logarithmic graph the correlation of CMV quantitation by CMV pp65 antigenemia and plasma PCR among 8 samples obtained from patients not on CMV treatment determined positive by both methods (R^2 ^= 0.68, P = 0.006).

To exclude any effect due to specific anti-CMV therapy, correlation analysis of pp65 AG and PCR results was only performed on 405 blood samples collected from patients not on ganciclovir, foscarnet or cidofovir therapy; the observed Kappa coefficient of agreement for both CMV assays was 0.304 (poor agreement). The correlation for both CMV assays were not significant among 43 out of 405 samples positive by both methods or by either methods (R^2 ^= 0.000144, p = 0.94). On the contrary, when both tests were positive, a significant correlation was found (R^2 ^= 0.68, p = 0.006) (FIG. 1). Plasma PCR assay detected a median of 948 copies/ml (range 438–22600 copies/ml), while pp65 Ag assay showed a median of 6 positive cells/200000 PBL examined (range 1–400 cells).

### Time to detection and disappearance of pp65 antigenemia and PCR in transplanted patients

Of the 38 patients, 28 (73.6%) developed positive pp65 Ag after a median of 41 days (range 0–100 days), while 15 (39.5%) developed a positive PCR after a median of 43 days (range 5–67 days) (p = 0.64, Student's t-test) from SCT (Table 4). In 16 patients, CMV was first detected by pp65 Ag alone (13 blood samples had 1 to 10 positive pp65 Ag cells, 3 blood samples had 40, 32 and 20 positive pp65 Ag cells, respectively), in 5 by both methods and in 6 by PCR assay alone (range 470–1710 copies/ml); one patient had CMV biopsy-proven intestinal disease without pp65 Ag and PCR assays positivity before CMV disease (Table 3). The median time necessary to obtain a disappearance of pp65 Ag and a negative PCR result was 7 days (range 7–49 days) and 24 days (range 7–42 days), respectively (p = 0.76) from the beginning of pre-emptive therapy.

**Table 4:** Interval from transplantation to detection of first positive CMV test of samples from 38 SCT transplant recipient

Test method	No. positive/total (% positive)	Time (days to positivity)			
			
		Mean	Median	Range	SD
Pp65 antigenemia	28/38 (73.7)	42.93	41	0–100	20.06
Plasma PCR	15/38 (39.5)	40.20	43	5–67	14.49

### Incidence of positive pp65Ag and plasma PCR after SCT

For transplanted patients, the frequency to have a positive pp65 Ag assay (28/38 patients, 73.6%) was significantly higher than a positive PCR assay (15/38 patients, 39.4%) (p = 0.003). When the type of transplant was analyzed, the frequency to have a positive pp65 Ag assay (21/30 patients, 70%) was significantly higher than plasma PCR assay (10/30 patients, 33.3%) (p = 0.013) in HLA-identical sibling, but was similar in unrelated donor transplant (pp65 Ag positive in 7/8 patients, PCR positive in 5/8 patients) (p = 0.375). The frequency of CMV active infection was also similar between HLA-identical sibling transplant recipients and unrelated donor transplant, demonstrated by both the pp65 Ag assay (21/30 patients, 70% vs 7/8 patients, 87.5%), respectively, (p = 0.653) and PCR assay (10/30 patients, 33.3% vs 5/8 patients, 62.5%), respectively (p = 0.223).

When the incidence of CMV reactivation was analyzed according to the severity of acute GVHD, patients with grade II–IV acute GVHD developed CMV infection at a frequency similar to those with grade 0–1 acute GVHD. The frequency of positive pp65 Ag was 63.6% in 11 patients with grade II–IV acute GVHD and 77.7% in 27 patients with grade 0–I acute GVHD (p = 0.62). Similarly, the frequency of positive PCR was 36.4% in 11 patients with grade II–IV acute GHVD and 40.7% in 27 patients with grade 0–1 acute GVHD (p = 0.99).

No differences between positive pp65 Ag and plasma PCR assays were also found when patients were evaluated according to the dose of acyclovir. The frequency of positive pp65Ag was 72.4% in 29 patients who received acyclovir at standard dose and 77.8% in 9 patients who received acyclovir at high dose (p = 0.90). Similarly, the frequency of positive plasma PCR assay was 34.5% in 29 patients who received acyclovir at standard dose and 55.5% in 9 patients who received acyclovir at high dose (p = 0.43).

### Patients with CMV diseases

Three patients developed intestinal CMV disease (7.9%): of these, one had negative pp65Ag and PCR assays before CMV disease, one had a concomitant positivity of both methods (400 Ag positive cells and 22600 copies/ml) and in the remaining patient only pp65 Ag was positive (7 positive cells) 1 week before the onset of CMV disease (Table 3).

The UPN 0136.1 patient (Table 3) was diagnosed of renal cancer on December 2002. The disease relapsed on May 2003 and the patient underwent allogeneic bone marrow transplantation on September 2003. Conditioning regimen included total body irradiation and fludarabine. GVHD prophylaxis was based on mycophenolate mofetile and cyclosporine. On day 39, the patient developed a grade II acute GVHD (skin) and therapy with prednisone 2 mg/kg/day was given. During the steroid treatment the patient developed diarrhoea and was admitted to hospital in the suspect of intestinal GVHD. On day 49, pp65AG (400 cells)and PCR (22600 copies/ml) on blood sample were positive. A colonoscopy demonstrated an hemorrhagic enterocolitis with viral inclusions in intestinal cells; immunohistochemical analysis was positive for CMV. The patient was diagnosed of CMV enteritis and therapy with intravenous ganciclovir 10 mg/kg per day was administered. In the following 4–6 weeks, pp65Ag and CMV DNA decreased (Table 3); nevertheless, there was no resolution of intestinal symptoms. The patient died for CMV disease associated with acute GVHD on day 111.

The UPN 0138.1 patient (Table 3) was diagnosed of philadelphia positive lymphoblastic leukemia on March 2002. On August 2003, the patient relapsed and underwent an unrelated donor bone marrow transplantation on November 2003. On day 14 after transplant, pp65AG was positive (7 cells), while plasma PCR was negative and therapy with foscarnet 180 mg/Kg/day was administered. Because of diarrhea, a colonoscopy was performed. By an intestinal biopsy, there was the evidence of viral inclusions, and the immumnohistochemical analysis was positive for CMV. Foscarnet was switched to intravenous ganciclovir because of renal failure. There was a rapid resolution of diarrhoea and the pp65AG was negative after the first week of treatment. On day 42, ganciclovir was stopped and the patient was discharged with oral acyclovir. One week later the patient had a positivity of both methods (pp65Ag:60 cells, plasma PCR:1230 copies/ml) for CMV without gastrointestinal symptoms. Therapy with ganciclovir was re-started, a progressive reduction of pp65 Ag (Table 3) and a relevant increase of CMV DNA (Table 2) were observed in the following weeks. The complete clearance of both pp65Ag and plasma PCR was observed following day+100. The patient died on May 2004 for JC virus leucoencephalitis.

The UPN 0133.1 patient (Table 3) underwent allogeneic bone marrow transplantation for multiple myeloma. On day 56, the patient developed diarrhoea, both pp65Ag and plasma PCR were negative. Intestinal biopsy was positive for nuclear inclusions and immunohistochemical analysis was positive for CMV. The patient started therapy with foscarnet 180 mg/kg/day with resolution of intestinal symptoms. Foscarnet was stopped on day 77. On day 100, only pp65Ag assay (2 cells) was positive and the patient did not complain any intestinal symptoms. Therapy with intravenous ganciclovir was started with disappearance of pp65Ag.

## Discussion

Despite major advances in treatment and prevention, CMV infections remain an important cause of morbidity and mortality in SCT recipients. Prophylaxis with ganciclovir during the first 100 days after transplantation results effective in preventing CMV disease in high risk patients, but is also associated with significant myelotoxicity, increased incidence of invasive fungal infection, and late CMV disease due to delayed CMV-specific T-cell response recovery [10,29,30]. Ganciclovir or foscarnet given pre-emptively to patients with documented active CMV infection (positive pp65 Ag and/or PCR), can spare a significant proportion of the population undergoing SCT from exposure to potentially toxic antiviral therapy. Therefore, a sensitive diagnostic test that reflects an active CMV infection, such as pp65 Ag assay, is essential for the success of pre-emptive therapy. In fact, using a pre-emptive strategy based on a low threshold for the pp65 Ag assay (any positive result) followed by a long ganciclovir therapy (until day 100 post-SCT), the incidence of early CMV disease was reduced to 3.8% [11], compared to 14% found in a different study using a cut-off value of three positive cells per 300.000 PBL examined [10]. In our study, the pp65 Ag-guided strategy for the initiating of pre-emptive therapy (any positive result) resulted in an incidence of CMV disease before day +100 of 7.8%, rate comparable to the recently reported incidences ranging from 0 to 16% [10,11,14,25]. Furthermore, our pp65 Ag-guided pre-emptive therapy, resulted in low frequencies of secondary episodes of active CMV infection (Table 2, 3) and zero episodes of late CMV disease were documented.

In our study, we prospectively compared the results of plasma PCR and pp65 Ag assay; two commercially available detection assays to monitor CMV infections in SCT recipients receiving a pre-emptive antiviral therapy initiated because of a positive pp65 Ag assay result. A clinical finding of our study is the slight earlier positivity, although not statistically significant, of pp65 Ag assay over quantitative plasma PCR assay, finding in agreement to those already reported by Boeckh et al. [25] and Boivin et al. [31]. An earlier study suggested that detection of CMV DNA in plasma might be equivalent to detection of CMV by pp65 Ag [21]. In the present study, however, despite the overall high sensitivity [17-21], PCR assay was not positive in all blood samples that were found to be positive by the pp65 Ag assay (Table 2, 3). Our study was not designed to compare plasma PCR vs pp65 Ag as the best parameter for starting pre-emptive therapy; therefore, no conclusions can be made on the clinical sensitivity of PCR assay, since pre-emptive therapy was based on pp65Ag assay.

Discordances between the positivity rates of the two assays were found in our study. This discrepancy may be explained: 1) by the choice of pp65 Ag assay for guiding antiviral therapy, which may have modified CMV viral load to be detected by plasma PCR; 2) the two methods target different markers of virus replication, detecting free virions in plasma and virus protein in PBL, with different levels of sensitivity [17-21]. In addition, most of the samples with discordant results were found to be pp65 Ag positive and plasma PCR negative. Our data are in agreement with those reported by Boivin et al. [31] and by Boeckh et al. [25], but are discrepant with those reported by Solano et al. [17]. The reasons for such a discrepancy are not clear since the protocol used to perform pp65Ag assay and the type of PCR appeared not to be substantially different from that followed in our study. However, diagnosis based on molecular methods for early detection of CMV DNAemia in solid organ transplant [32,33] and in bone marrow recipients [34] appears to correlate better with active viral replication and with prediction of CMV disease than pp65AG. However, it should be mentioned, that, CMV disease may still develop in patients who have negative pp65AG or undetectable DNA [1].

Because a low CMV viral load is highly significant in the context of SCT, the primary objective should be on improving sensitivity [26,35]. Therefore, when in our study detection of ≥1 antigen positive cell and ≥400 CMV DNA copies/ml (lower detection limit of Cobas Amplicor CMV Monitor test) were defined as positive results, the pp65Ag assay showed 73%/100%/100%/95%, for sensitivity/specificity/positive predictive value/negative predictive value, using any positive test (either pp65 Ag or PCR assays) as the reference. Boeckh et al [25] demonstrated that detection of CMV DNA in PBL by PCR was the most sensitive method, followed by the pp65Ag assay, detection of CMV DNA in plasma and viremia. Their antigenemia assay showed 62%/88%/45%/94% for sensitivity/specificity/positive predictive value/negative predictive value, using plasma PCR as the reference. Yakushiji et al. [36] compared the results of pp65 Ag assay with those obtained by plasma real-time PCR; the pp65 Ag assay showed a sensitivity of 55.4% and a specificity of 95.5% using the real-time PCR as the reference. The sensitivity of our pp65 Ag assay was similar to those obtained by Boeckh et al [25], Boivin et al [31], and Yakushiji al. [36].

As the pp65Ag assay was found to be positive earlier than PCR, it became negative faster after initiation of antiviral therapy, although these differences did not reach statistical significance. The finding of faster clearance of pp65 Ag compared to plasma CMV DNA after treatment has been observed in the context of SCT [17,31]; it is interesting to note that patients who continued to be plasma CMV DNA positive after conversion of the pp65Ag (from positive to negative result) did not progress to CMV disease. Therefore, pp65Ag assay is suitable for monitoring the efficacy of anti-CMV therapy.

In particular, the kinetics of pp65AG and plasma DNA assays in a patient with CMV disease (UPN 0.138.1) (Table 3), show a disconnection between pp65Ag and PCR as the pp65Ag values are decreasing (from 60 to 4 cells) from days +49 to +63 while CMV DNA (from 1230 to 16700 copies/ml) is actually increasing. After 10 weeks of ganciclovir therapy the patient recovered from CMV infection. We have not a definitive explanation for this phenomenon.

In our study, the probability of patients to have a pp65 Ag positive assay was higher than to have a PCR positive assay. When we analyzed this finding, pp65 Ag positive result was statistically more frequent than PCR result in patients receiving an HLA-identical sibling transplant. However, 11 of these 21 patients had a positivity only for pp65 Ag with a low level of positive cells (median 2 positive cells, range 1–5). This suggests a CMV late phagocytosis by PBL, despite the absence of virus replication [37].

Interestingly, a trend of more frequent CMV infection (either pp65 Ag assay or PCR assay) was shown in unrelated donor transplant recipients than HLA-identical sibling patients, while patients with acute GVHD II–IV grade did not develop more CMV infection than those with grade 0–1 acute GVHD. These findings could be explained by the lower number of unrelated patients compared to related who entered in our study. Further, in our study, statistical analyses may be influenced by the small number of patients, by the preexisting heterogeneity of conditioning regimens, GVHD prophylaxis, hematological diseases and donor type.

The choice of the ideal sample material for PCR (plasma, serum, leukocytes, or whole blood), remains to be elucidated. Furthermore, viral loads obtained in whole blood, where higher than those measured in plasma, serum, PBL and peripheral blood mononuclear cells [21]. Although the use of plasma may lead to a loss of sensitivity as compared with leukocytes, it detects only free virions indicative of active viral replication with a higher clinical relevance [1,23,24]. Thus, it could minimize over-treatment and discriminate between active infection and latent infection. Although comparative results of different methods and samples materials have been reported, it seems difficult to draw definitive conclusions as these different methods have been evaluated in different patient population (recipients of SCT or solid organ transplant, acquired immunodeficiency syndrome) [15,21,23,24,38], as well as the choice of primers may also influence the sensitivity of PCR. Recently, Boeckh M et al [39] developed a highly sensitive quantitative plasma PCR assay using two primers (UL55/UL123-exon 4); this assay was more sensitive than pp65 Ag assay and single-primer plasma PCR assays (UL125 alone, UL126 alone).

## Conclusion

Both pp65Ag and quantitative plasma PCR assays (COBAS AMPLICOR CMV MONITOR) are useful in early detection of CMV infection and prevention of CMV disease after allogeneic SCT. Moreover, in the three patients who developed CMV disease, PCR anticipated the onset of CMV disease in one patient, while pp65 Ag in two, showing that both tests are useful in diagnosing CMV diseases. Lower thresholds should be probably adopted for quantitative assays in the context of SCT, because small amounts of CMV load require immediate therapy. Therefore, further studies are required to determine the diagnostic value of each method to guide pre-emptive therapy by the most clinically suitable method in SCT recipients.

## Abbreviations

CMV: Cytomegalovirus,

pp65Ag: pp65 antigenemia,

SCT: stem cell transplantation,

PCR polymerase chain reaction,

PBL: peripheral blood leukocytes,

GVHD: Graft-versus-host disease,

CyA: Cyclosporin-A,

MTX: Methotrexate,

PDN: Prednisone,

PMN:polymorphonuclear cells

PLTS: platelets

MMF: micofenolato mofetil

ND: not done

NE: not evaluable

## Competing interests

The author(s) declare that they have no competing interests.

## Authors' contributions

All the authors contributed substantially to the study. GG and PDF designed the study, contributed to data analysis and wrote the manuscript. AP collected the data, contributed to data analysis and drafted the manuscript. AV contributed to design of the study and in writing the manuscript. AS performed the statistical analysis. LC, TD, AT, and LC collected the data. AC and MC conducted the laboratory studies. SA and PM provided critical comments for the manuscript. All authors read and approved the final manuscript.

## Pre-publication history

The pre-publication history for this paper can be accessed here:


